# Transient motion of the largest landslide on earth, modulated by hydrological forces

**DOI:** 10.1038/s41598-021-89899-6

**Published:** 2021-05-17

**Authors:** Gökhan Aslan, Marcello De Michele, Daniel Raucoules, Severine Bernardie, Ziyadin Cakir

**Affiliations:** 1 Natural Risk Department, BRGM-French Geological Survey, 3 Claude-Guillemin, 45060 Orléans, France; 2 Department of Geological Engineering, ITU, 34467 Istanbul, Turkey

**Keywords:** Climate sciences, Hydrology, Natural hazards

## Abstract

Sea-level rise of the Caspian Sea (CS) during the early Khvalynian (approximately 40–25 ka BP) generated hundreds of giant landslides along the sea’s ancient coastlines in western Kazakhstan, which extended hundreds of kilometers. Although similar landslides have been observed along the present-day coastlines of the CS in the area of a prominent high escarpment, it remains unclear whether some of these ancient landslides are still active and whether the movement is slow or catastrophic, as previously suggested. The present study is the first to show evidence proving that the geomorphic responses to sea-level changes of the CS that were triggered in the Pleistocene are currently active. Using interferometric synthetic aperture radar (InSAR) data, we show that one of these giant landslides occurring along the western shore of the Kara-Bogaz-Gol (KBG) lagoon of the CS presents active transient motion, which makes it the world’s largest active landslide reported thus far. Extending more than 25 km along the eastern coast of the inundated KBG depression in a N–S direction with maximum landward expansion of 5 km from the shoreline to the flat Ustyurt Plateau, this landslide conveys ~ 10 × 10^9^ m^3^ rocks toward the lagoon at a rate of ~ 2.5 cm/year. This event releases a nearly episodic aseismic moment of 6.0 × 10^10^ Nm annually, which is equivalent to the response of an Mw 5.1 earthquake. We analyze the present-day evolution of this giant coastal landslide at high temporal and spatial resolutions using Sentinel-1 radar images acquired on descending and ascending modes every 12 days between 2014 and 2020. Modelling with elastic dislocations suggests that the KBG landslide was accommodated mostly by a shallow basal décollement with a nearly horizontal listric slip plane. Moreover, our analysis reveals week-long accelerating slip events at changing amplitudes that occur seasonally with slow, lateral spreading rather than sudden catastrophic motion. A strong correlation between the episodic slip events and seasonal water-level changes in the KBG lagoon suggests a causative mechanism for the transient accelerating slip events. Although water-level changes are widely acknowledged to trigger transient motion on a land mass, such movement, which is similar to a silent earthquake, has not been observed thus far at this mega scale; on an extremely low-angle detachment planes at < 5° with modulation by sea-level changes. This study suggests that present-day sea-level changes can reactivate giant landslides that originated 40–25 ka.

## Introduction

Most giant terrestrial landslides > 10^8^ m^3^ usually occur in the steepest, deeply incised, and formerly glaciated landscapes of the world^1^, in addition to tectonically active mountain belts, flanks of volcanoes, and large escarpments^2^. Nearly two-thirds of these kilometer-scale gigantic slope failures are triggered by catastrophic events such as ground shaking from strong earthquakes, volcanic eruptions, and heavy rainstorms^3^. Recent observations have shown that some giant landslides, particularly those generated by Caspian Sea (CS) transgression in the late Pleistocene, can occur on a very gently inclined slip surface and in extremely low-relief landscapes far from active mountain belts^3–5^. These landslides are believed to have been modulated by sea transgression/regression cycles^3^.

The geomorphic response to sea-level changes on sea coasts, particularly in the CS, remains enigmatic. It is widely accepted that sea transgression in the late Pleistocene during the Early Khvalynian (approximately 40–25 ka BP) inundated vast portions of the low-lying semi-desert of western Kazakhstan/eastern Turkmenistan to form highstands^3^. Cliffs cut during the highstands generated the prominent escarpment that presently surrounds the Kara-Bogaz-Gol (KBG) lagoon of the CS, which intersects the area of a giant landslide. According to its geomorphic features, this slope failure is considered to be a fossil structure that once mobilized rock volumes > 10^8^ m^3^ along the basal failure planes with gradients as low as ~ 5°^3^. Previous studies reported that this type of catastrophic slope failure occurred mostly during the Pleistocene CS water-level highstands. However, whether some of these landslides were reactivated or whether they originated during the Holocene remains unknown, owing to a lack of knowledge of the slope failure’s present-day dynamics.

Slope instability, a common form of ground failure in coastal areas, poses a high risk to local infrastructure and public safety. The movement associated with these events is dynamic in both time and space and is closely linked to the stochastic nature of the environment such as the geology, geomorphology, and vegetation, as well as external disturbances such as climate change, earthquakes, and heavy rainfall^6,7^. Incomplete information of landslide surface displacement limits our ability to identify the physical and environmental characteristics that contribute to an area’s mass wasting potential and to calculate the driving and resisting forces occurring in a geological system^7^. Landslide displacement time series are usually considered to be complex non-linear phenomena^8,9^. Continuous monitoring and spatio-temporal analysis of the landslide deformation field can provide an important basis for reducing its damage potential. However, landslide detection in remote and inaccessible areas can be highly challenging when using conventional methods based on in situ measurements, which are inevitably subjective, cumbersome, time consuming, prone to error, and particularly difficult to perform. For these reasons, modern applications of multiple remote sensing technologies including synthetic aperture radar (SAR), optical methods, and light detection and ranging (LIDAR) measurement have gained significant interest in the last decades as complementary data sources relative to traditional mapping and monitoring methods.

Landslides occurring in remote locations far from infrastructure and human settlements generally pose little threat to human life. Despite their reduced impacts on the population and related economic damages, however, updating of pre-existing landslide inventory maps is necessary to better understand the kinematics of the landslides in a context in which climate change might alter the kinematics of the phenomenon. The landslide examined in the present study falls within this perspective. In particular, this study analyzes the present-day surface deformation of a giant coastal landslide characterized by slow movement velocity on a scale of centimeters per year^10^ associated with water-level change in the KBG lagoon. We determine that this coastal slope movement has evolved in response to present-day hydrological dynamics related to a changing climate.

Landslide mechanisms involve a broad range of mass-wasting processes exhibiting a variety of behaviors in response to a variety of environmental factors^11^. From a mechanical perspective, the changes in hydrological conditions in and around a landslide area are key factors in the pore water pressure buildup in the soil structure. This can cause a critical reduction in the soil’s shear strength provided by matric suction. The water infiltration process into a hillslope can have several origins such as rainfall, snowmelt, anthropogenic irrigation, and even atmospheric tide^12^. In a comprehensive review of landslide hydrology, Bogaard^13^ addressed the influence of hydrological processes in triggering landslides from both earth science and soil mechanics perspectives. Landslide hydrology is an interdisciplinary science and requires a close cooperation of experts from various fields such as geology, geophysics, geotechnics, and hydrology as well as measurement disciplines such as geodesy and remote sensing.

In recent years, various time series SAR techniques have been successfully employed to detect slope instability in relation to hydrological force as a potential triggering factor^7,14–17^. The available SAR archive in this landslide area is covered by Sentinel 1A/B data over one ascending and two descending satellite passes completed between 2014 and 2020. In this study, we compute the time series of the ground deformation field over the study area using the Stanford Method for Persistent Scatterers (STAMPS) package^18,19^. We then combine the results to compute the vertical and horizontal components of the surface deformation field over the landslide and its surrounding area. In addition, the patterns of observed coastal landslide deformation signals along the 20-km-long strip of the eastern bank of the KBG are interpreted in a spatio-temporal extent and are modeled. To the best of our knowledge, surface deformation monitoring along the banks of the KBG lagoon has not been performed previously.

## Study area

**Figure 1:**
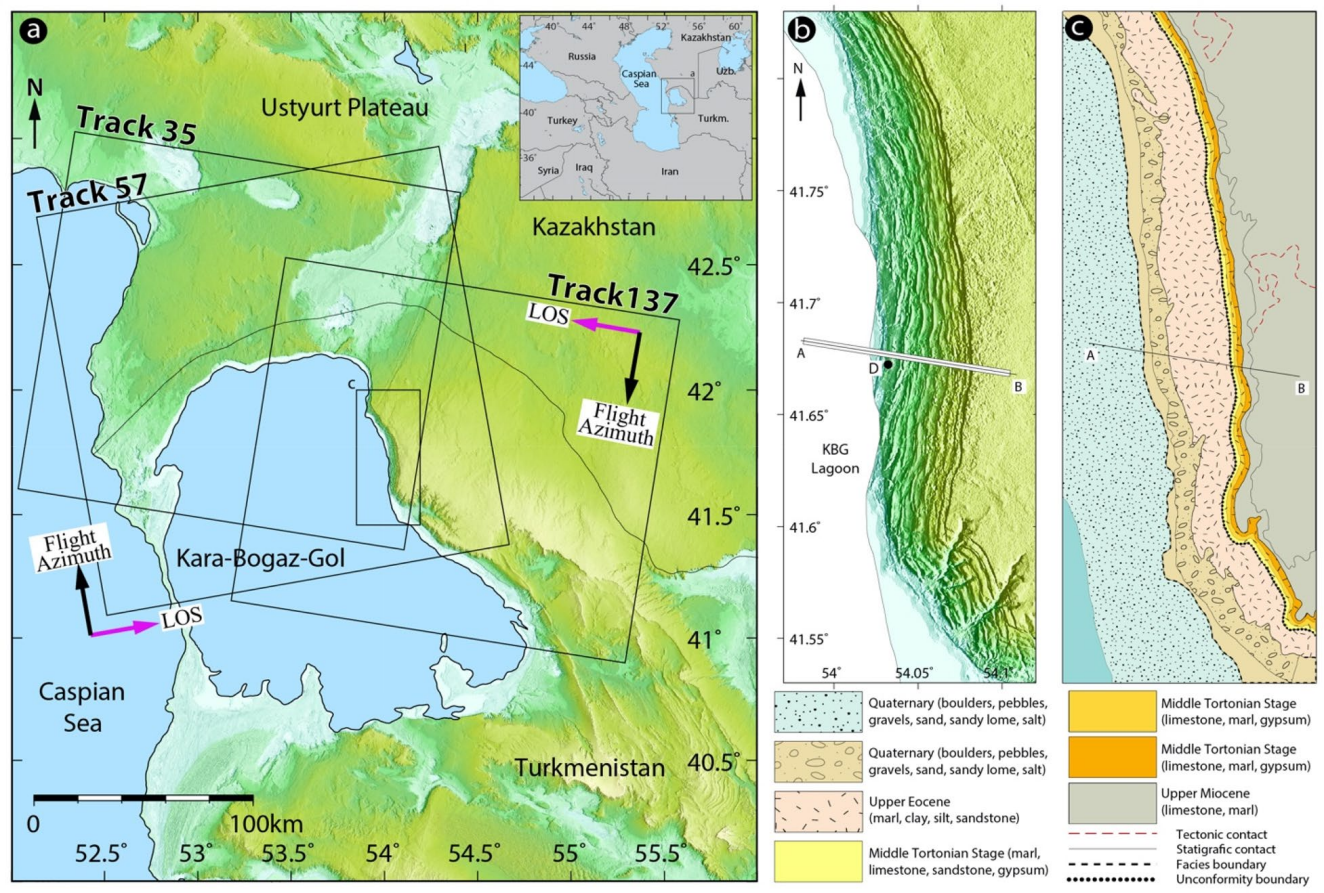
SAR data coverage and geological map of the study area. (**a**) Map showing the location of the study area. The inset map shows the location of the CS in relation to Asia Minor and Central Asia. Sentinel 1-A/B SAR data coverage is overlain on the 30 m shaded topography (ALOS (AW3D30) DEM data). Rectangles labelled with track numbers (Ascending tracks 57 and descending tracks 35 and 137) indicate the coverage of the IW SAR images. Magenta and black arrows indicate the satellite’s LOS look and flight directions, respectively, and the black rectangle indicates the selected study area. (**b**) Topographic view of the eastern bank of the KBG lagoon including the stepped topography caused by lateral spreading of the plateau toward the lagoon. (**c**) Schematic geological map and lithological descriptions taken from the OneGeology Platform (http://www.onegeology.org); the A–B profile along the landslide path is shown in Fig. 5. This figure was created with Adobe Illustrator CS6 ver. 16 (http://www.adobe.com/products/illustrator.html).

Known as the earth’s largest inland water body both in area and volume, the CS is located in a large continental depression about 27 m below mean sea level with no surface outlets, which makes its water level notably dependent on the balance among climatic variations (Fig. 1)^20^. The water-level change in the CS has undergone substantial fluctuations over the past several hundred years with a drop of 3.5 m from 1900 to 1977 and a rise of > 2 m from 1977 to 1994 (e.g., Refs.^20–23^). The causes for this fluctuation over the entire historical period remain poorly understood, although cyclic behavior is apparent^24,25^. In such an enclosed basin, this fluctuation is controlled mainly by river flow into the sea, precipitation, and loss from evaporation and discharge to the KBG Bay^26^.

The KBG, which literally translates to Black Strait Lake in Turkmen (bay–zaliv in Russian literature), is a large and quite shallow lagoon that is 1.8 × 10^4^ km^2^ in area and a few meters deep. It is separated from the CS by a narrow rocky ridge (Fig. 1) and has a very narrow inlet through which Caspian water flows and cascades down into it^27^. This connection makes the water level of the KBG closely related that of the CS, although the enormous natural evaporation rate of the former causes its water level to remain lower than that of the latter^27^. Such a difference in water level between the CS and the bay leads to water flows at speeds of 50–100 cm/s from the CS through a narrow strait into the KBG, where it evaporates^28^. In addition to the natural climatic and hydrologic causes of water level change in the KBG, anthropogenic factors led to dramatic decreases in the water level by a dam constructed in 1980, in order to retard the fall of the CS water level which led to complete desiccation of the lagoon and unintentional catastrophic drying^26,27^. In 1984, four years after the dam was built, the seawater was directed into the bay by pipes at a rate of 1.6–1.8 km^3^/year, which lasted for nearly a decade. However, such restricted water supply of seawater from the CS did not make any salient improvement in the hydrological conditions in the KBG considering its high evaporation rate^26^. After the dam was dismantled in 1992, the lake was filled completely with CS water until its equilibrium state was reached^26^. The various aspects of the KBG water body have been discussed extensively in previous research (e.g., Refs.^25,27^).

The landslide complex along the eastern shore of KBG comprises numerous slices that form a staircase morphology from the shoreline to the Ustyurt Plateau, at − 30 to ~ 300 m in elevation (Fig. 1b). Rising groundwater tables owing to CS water-level increases played a major role in activation of such mega landslides not only during the Holocene but also in the Pleistocene epoch. This is evidenced by similar landslides and rock flows observed along the paleo-coastlines inland within the western Kazakhstan’s Caspian Depression 200–300 km to the northeast of the study area^3^.

## Water level oscillations of KBG lake

To more effectively illustrate the dynamics of the KBG water level, we should first highlight the relationship between the CS and the KBG. As discussed in the previous section, the CS level has shown a dramatic decline during the past several hundred years. In their comprehensive study, Chen et al. provided valuable insight into the dynamics of lake-level fluctuations of the CS^23^ by compiling information on the water-level changes from different data sources and models. They reconstructed the time series of CS water-level change by using historical records obtained from tide gauge measurements over the period 1840–2000 and from continuous satellite altimeter observations from TOPEX/Poseidon and Jason-1-2-3 missions over two decades since 1992 in combination with records of precipitation and drainage into the sea from rivers as well as the estimated evaporation from a climate model. The CS water level has dropped almost 7 cm annually from 1996 to 2015, for a total of nearly 1.5 m^23^. According to their analysis, the high evaporation rate over the CS, which was linked to increased surface air temperature likely as a result of climate change, was the primary cause of the water-level drop in the lake^23^.

The dramatic water-level drop since the 1930s has been attributed to fluctuations in the discharges of the Volga and Kama rivers as a result of combined natural impacts, such as rainfall reduction over the catchment area, and anthropogenic factors, such as the construction of cascade reservoirs^23,28^. Following this dramatic water-level drop in the CS, Soviet scholars developed various projects to rescue the CS and reverse the serious impacts on the ecosystems and the coastline. Among them, the easiest and most cost-effective project involved stopping the water discharge into the KBG ^20^. In 1980, the narrow strait connecting the KBG lagoon to the CS, with dimensions of 110–300 m in width and 10–12 km in length, was dammed to prevent water from flowing into the KBG basin. In response to this anthropogenic intervention, the KBG had vanished completely by November 1983^29^.

Since the 1930s, the discharge from the CS into the KBG significantly changed in relation to the sharp fall in the level of CS water^26^. The supply of seawater to the lagoon decreased from ~ 37 km^3^/year in 1884 to 6 km^3^/year in 1939 and disrupted the water balance. The decrease in the water volume of KBG Bay until 1980 is attributed to long-term decreases in the CS water level^26^. The volume of inlet water into KBG Bay continued to decrease until the dam cutting off the bay was dismantled in June 1992. Following the opening of the narrow strait, the water level began to rise quickly within a few years^26^.

## Water balance of KBG and CS

After complete dismantling of the dam in 1992, the CS water began to flow freely into the saline water of the KBG basin to eventually establish the current balance and a new hydraulic regime between the sea level and that of the lagoon. According to the decadal radar altimeter sea surface measurements (Supplementary Fig. 1), the difference between levels in the CS and KBG is between 0.30 and 0.70 m depending on the season. This balance between the water levels in the sea and bay was established during the last two decades. The seasonal water level difference is minimum during winter, at 0.3–0.4 m, and increases to 0.7–0.8 m during summer owing to the more intensive evaporation occurring in the lagoon^26^. In recent decades, the KBG Bay water-level evolution with characteristic seasonal oscillation has shown a clear decreasing tendency with a rate of 10–12 cm/year, as observed from TOPEX/POSEIDON/Jason 1-2-3 altimetry missions. Currently, the fluctuation in water level occurs at 28.5 m in elevation.

## General tectonic/geologic setting of the study area

The Transcaspian Depression is of great economic importance for the region’s countries owing to numerous oil and gas reservoirs (e.g., Ref.^30^); however, the geological puzzle of the Caspian region remains underexplored. Detailed geological data of the eastern side of the CS were collected during the 1960s and 1970s by Soviet teams, particularly in the Greater Balkhan Uplift and the Tuarkyr Uplift. It is believed that Soviet marine geophysics disciplines such as magnetic and electrical fields and seismic gravity originated through studies of the CS^31^. A detailed study of the main geological structures, characteristic features of tectonic evolution, and seismicity of the CS and its adjacent areas has been conducted by the Institute of the Lithosphere of Marginal Seas of the Russian Academy of Sciences (RAS)^30^ based on numerous geophysical surveys and borehole investigations.

Essentially, the CS represents a remnant of the ancient Tethis Ocean or, more precisely, its Paratethis Bay^31^. The CS is a vast land-locked water reservoir that exhibits close meridional stretching in an almost N–S direction crossing various major latitudinal structural elements. These structural elements consist of crustal blocks of different ages ranging from the Precambrian North Caspian Depression to the north, which belongs to the Russian platform, to Alborz Alpine folding in the central and southern parts of the basin. The study area lies within the Turan platform, the main tectonic unit in the region, which is bounded by the southwestern part of the Altaids to the north and the Tethyside units to the south^32^. The tectonic nature of the second-order units composing the Turan platform and their contact relationship has been defined on the basis of large amounts of subsurface data collected by drilling and geophysical methods^33–36^.

Despite the presence of a coherent unit, the Turan block is actually a mosaic of micro blocks^37^. The Turan platform is characterized by uniform echelon arrangement of magnetic and gravity anomalies oriented NW–SE, which are associated with the second-order units within the Turan domain^32^. These second order units, from NE to SW, are composed of Bukhara, Chardjou, Karakum-Mangyshlak, Tuarkry, and Karabogaz (Supplementary Fig. 2).

**Figure 2:**
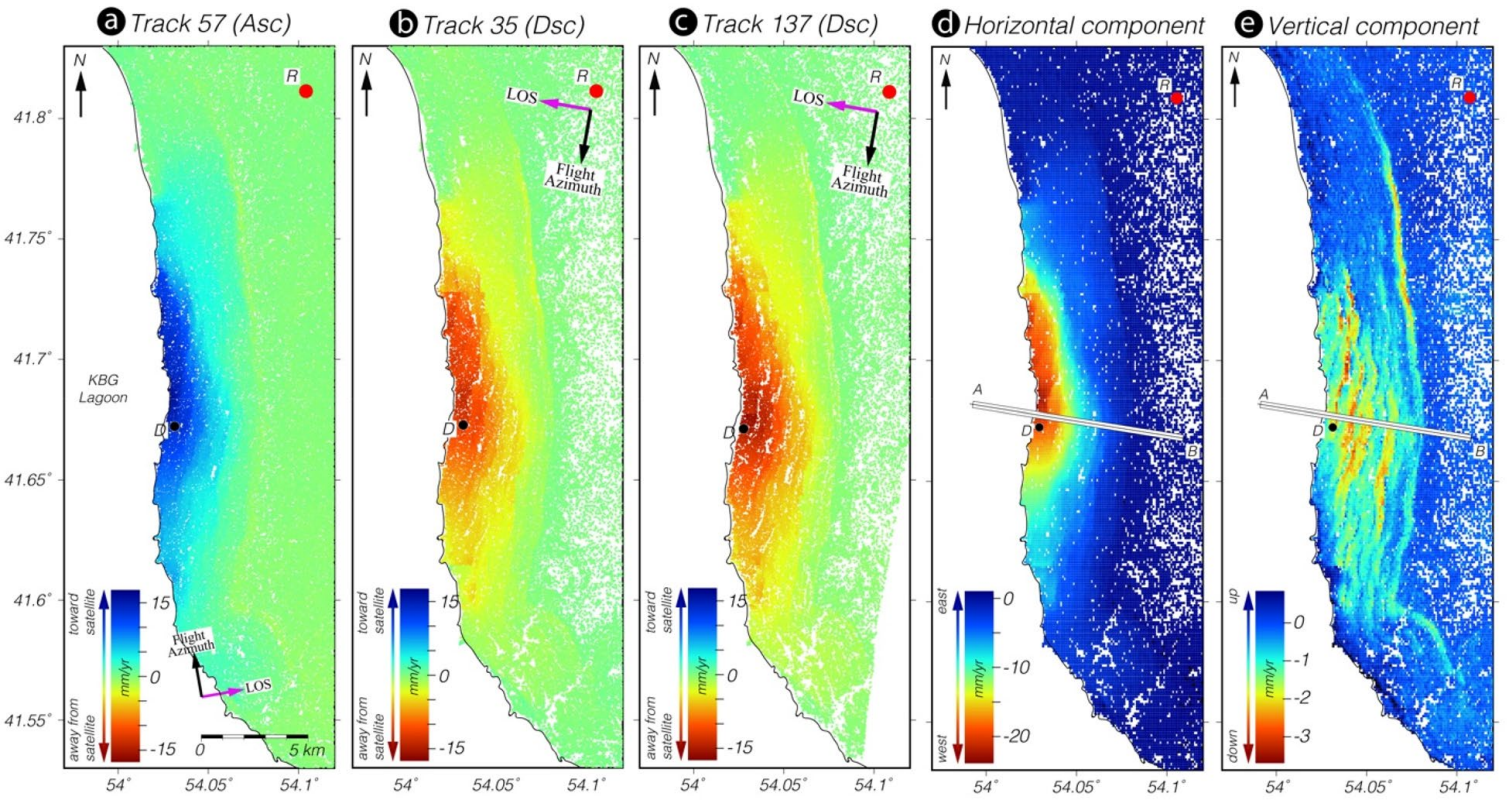
Mean LOS velocity fields of the KBG landslide and deformation decomposition into 2D displacement rates. Mean LOS velocity fields for the period 2014–2020, according to Sentinel 1A/B (**a**) ascending track 57 and descending tracks (**b**) 35 and (**c**) 137 obtained from the InSAR PSI time series analysis. Negative velocities (cold colors) represent ground motion toward the satellite; positive velocities (warm colors) represent motion away from the satellite. The mean velocity value of the PSI points within the solid black point (D) in (**a**), (**b**), and (**c**) is used to illustrate the temporal evolution of the landslide (Fig. 4) deformation with respect to the reference PS points within the red point (R) in the northwest region, which is considered to be a stable area. The red point (R) shows the area to which all InSAR velocities is referenced before decomposition. (**d**) Horizontal and (**e**) vertical velocity fields inverted from the LOS velocity fields of the Sentinel satellite tracks used in this study (2014–2020). Profile A–B is used to plot the vertical and horizontal velocities and elevations together with the geological cross-section shown in Fig. 5. The mean velocity value of the PS-InSAR points within the solid black point (D) in (**d**) and (**e**) is used to illustrate the temporal evolution of the landslide deformation (Fig. 4) with respect to the reference PS points within the red point (R) in the northwest region, which is considered to be a stable area. This figure was created with MATLAB (R2011a). (https://www.mathworks.com/products/matlab.html).

The detected mega landslide activity is located in the center of the NW-striking Tuarkyr unit (Fig. 2), which is geologically heterogeneous. According to data from borehole logging performed within this unit^33^, the arc massif is identified by granites and andesites. The Jamal well (dz), which is located in the southeastern Tuarkyr unit, penetrated 300 m of tuffaceous, poorly sorted sandstones, conglomerates, and siltstones as well as felsic tuffs and 30-m-thick andesitic lava flow^32,36^. The other two wells, located in the northwestern region of the unit, penetrated late Paleozoic granitic intrusions and cuts through slates of unknown age^36^. On the basis of these borehole records^36^, the predominant rock types are marine siltstone, shale, and poorly sorted conglomerates containing clasts of schists, cherts, and magmatic rocks. Geologically, the study area is characterized by a sequence of tilted layers of Eocene marls, clay, silt, and sandstones; mid-Tortonian gypsum, marl, and limestone; and Pliocene limestone, chalk, and sandstone.

## InSAR observations

Persistent Scatterer InSAR (PSI) time series analysis of 354 Sentinel IW SAR images on the three tracks revealed the spatio-temporal evolution of the landslide complex, as detailed in the “Methods” section. The mean line-of-sight (LOS) maps and vertical and horizontal maps calculated from Sentinel 1 data show that the land mass is sliding westward toward the sea dominantly in a horizontal direction at a maximum rate of 3 cm/year (Fig. 2). As shown in the vertical velocity map in Fig. 2e, relatively higher rates of vertical motion (yellow to red areas) are localized along narrow strips of terraces formed by secondary slip planes that likely accommodated the internal deformation of the landslide mostly by rotation (Fig. 2e). The overall vertical motion, depicted in the figure by turquoise coloring, is only about 1–2 mm/year. This implies that sliding must be occurring on a slip plane with an extremely shallow dip, which is known as a décollement. As described in the Methods section, modelling with elastic dislocation on a décollement with a dip of ~ 2° near the shore explains most of the motion with a root-mean-square (RMS) error of about 1 mm/year and predicts maximum displacement of 6 cm/year (Fig. 5; Supplementary Figs. 4–6).

## Time-dependent landslide deformation

**Figure 3:**
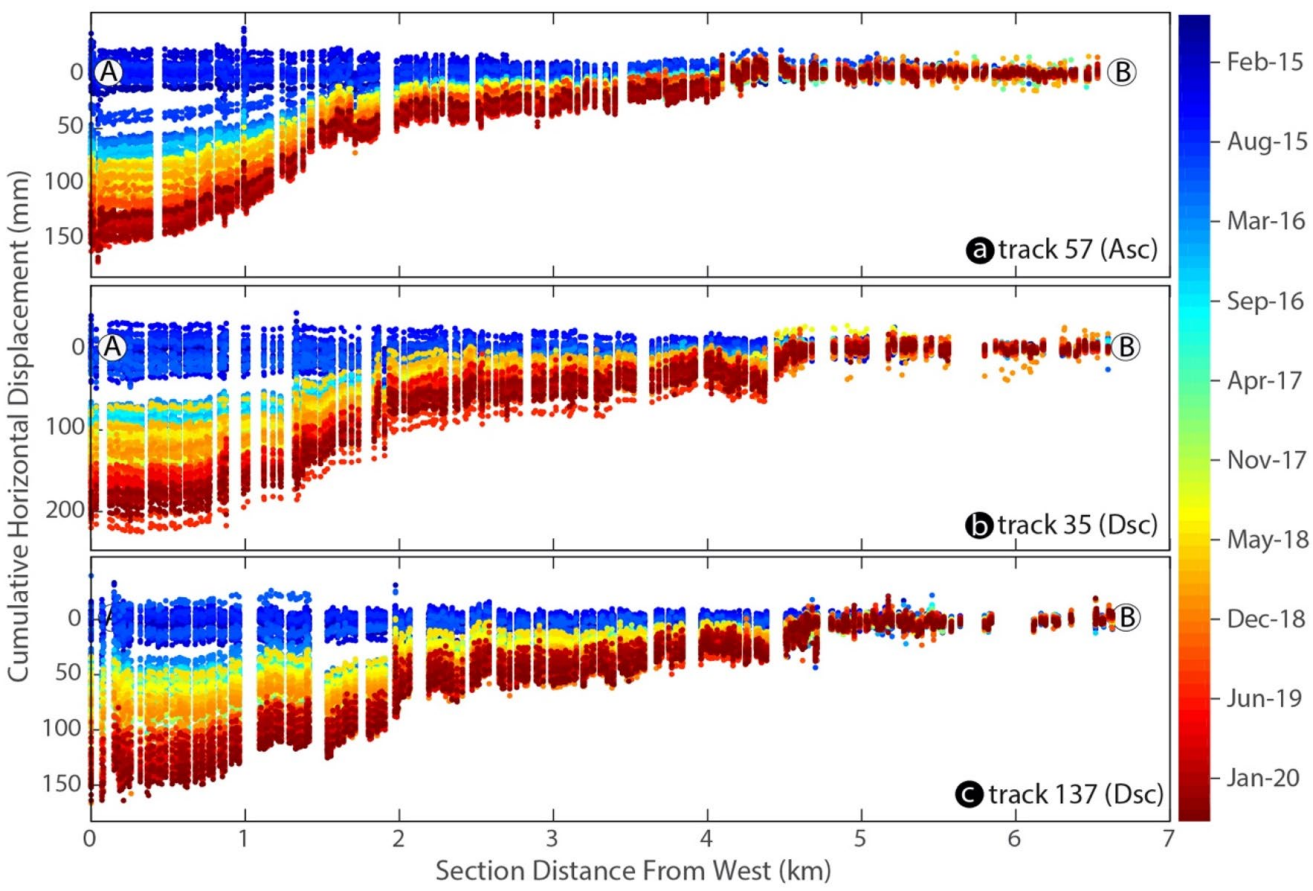
Profiles of InSAR LOS displacement time series (2014–2020) obtained from each track used in this study over the assumed stable area. The profile is marked by the swath profile A–B presented in Fig. 2d. This figure was created with MATLAB (R2011a). (https://www.mathworks.com/products/matlab.html).

The spatio-temporal evolution along the profiles shown in Fig. 3 indicates that the sliding is characterized by a persistent motion as well as transient, accelerating creep events with varying amplitudes. We observed the most significant transient motion, represented by a distinct jump on the profiles, in April 2016 on each track.

**Figure 4:**
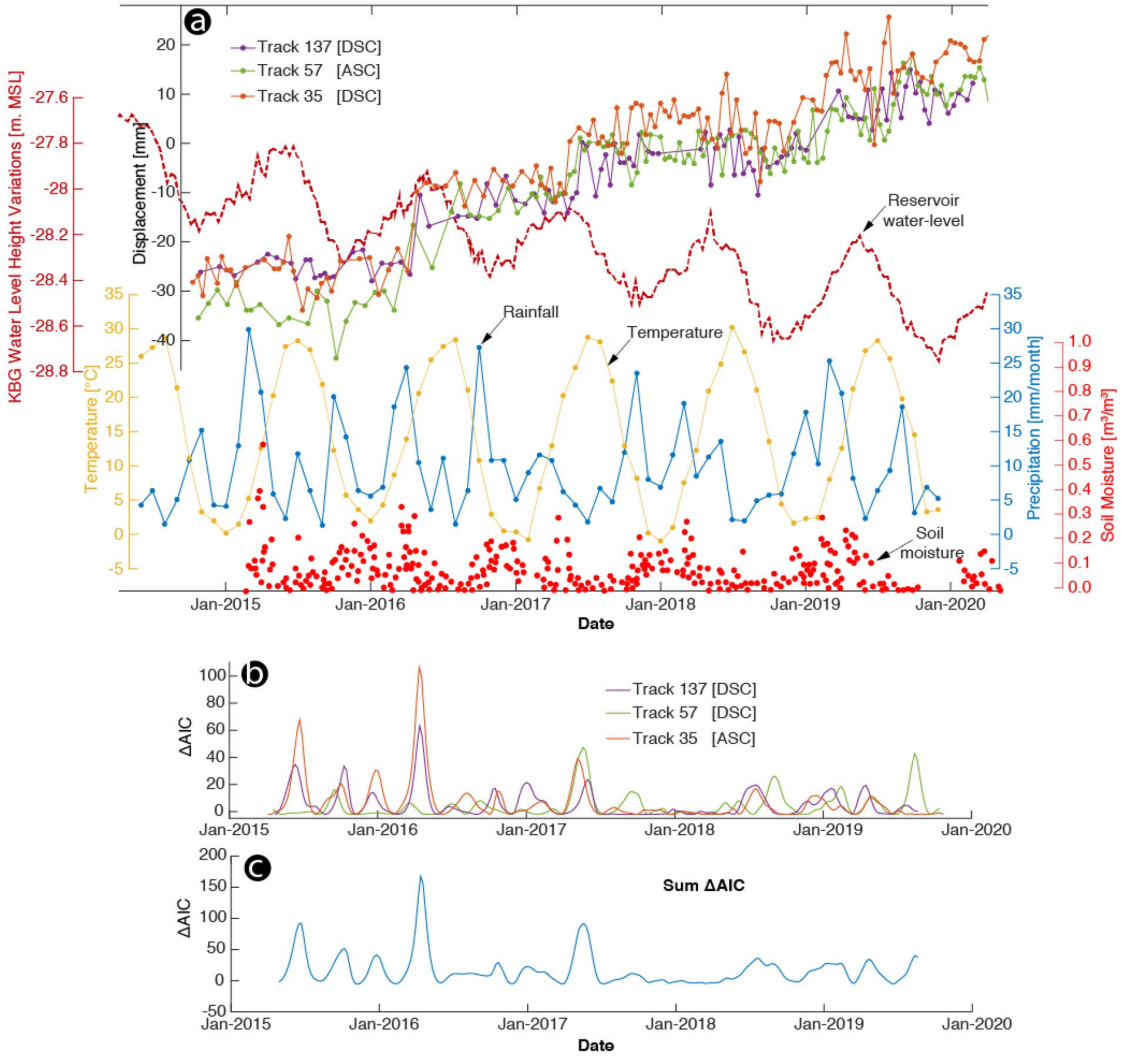
Landslide motion estimated from Sentinel-1 ascending track 57 (green) and descending tracks 137 (purple) and 35 (orange) used in the InSAR time series analysis compared with hydrological and atmospheric parameters derived from multiple satellite sensors. (**a**) InSAR-derived multi-annual landslide motion with week-long transient accelerating burst-like creep events, KBG lake water level height variations in meters above mean sea level (burgundy), temperature (°C; yellow), precipitation (mm/month; blue), and soil moisture (m^3^/m^3^; red). Velocity time series were projected onto the horizontal direction for better comparison. (**b**) ΔAIC between linear landslide sliding rate models with and without transient acceleration events computed for each track independently. $\Delta {\text{AIC}}$ is positive when the transient model is preferred. (**d**) Summation of all $\Delta {\text{AIC}}$ highlighting one major transient event. This figure was created with MATLAB (R2011a). (https://www.mathworks.com/products/matlab.html).

To more effectively illustrate the temporal evolution of this deformation, we calculated a time series of all points in the selected unstable area, represented in Fig. 2 as the solid dot (D). The results are shown in Fig. 4. The landslide displacement showed a well-marked stepped trend. The correlation of these prominent transient movements with the seasonal water-level change in the reservoir of the KBG landslide indicates that it could be the operative trigger mechanism. This is most likely associated with a change in pore water pressure. Despite the strong correlation between the multi-year precipitation rate and soil moisture over the study area, this desert region receives a little precipitation. Therefore, it is difficult to predict the contribution of fluid pressure evolution owing to surface water infiltration.

This slow and accelerating dynamic motion is characterized by transient or persistent sliding at low velocities and is affected by a certain level of noise, at about 3 mm/year. To test its statistical validity, we computed the Akaike information criterion (AIC^38^), as is currently performed for analysis of the Global Positioning System (GPS) time series (e.g., Ref.^39^). Details of this procedure are given in the “Methods” section. We observed the transient displacement of the landslide body every year except for 2018, and all had different slip amplitudes. The observed $AIC$ peaks from all three SAR tracks were temporally correlated with each other. We observed that the transient motion highlighted by the InSAR time series occurred every year in April/May, when the KBG reservoir water level reached its seasonal maximum.

In addition, the summed $\Delta AIC$ showed that the periodic displacement declined in amplitude during the observation period. This could be associated with the persistent water-level decline in the KBG lake since 2006 (Supplementary Fig. 1).

The Sentinel deformation time series revealed seasonal transient behavior in response to seasonal multi-year changes in the water level. Although these processes are the results of the hydro-mechanical relationship between the moving material and hydrological regime, the landslide-related ground displacement can also be indirectly considered as a result of climate-driven cycles.

**Figure 5:**
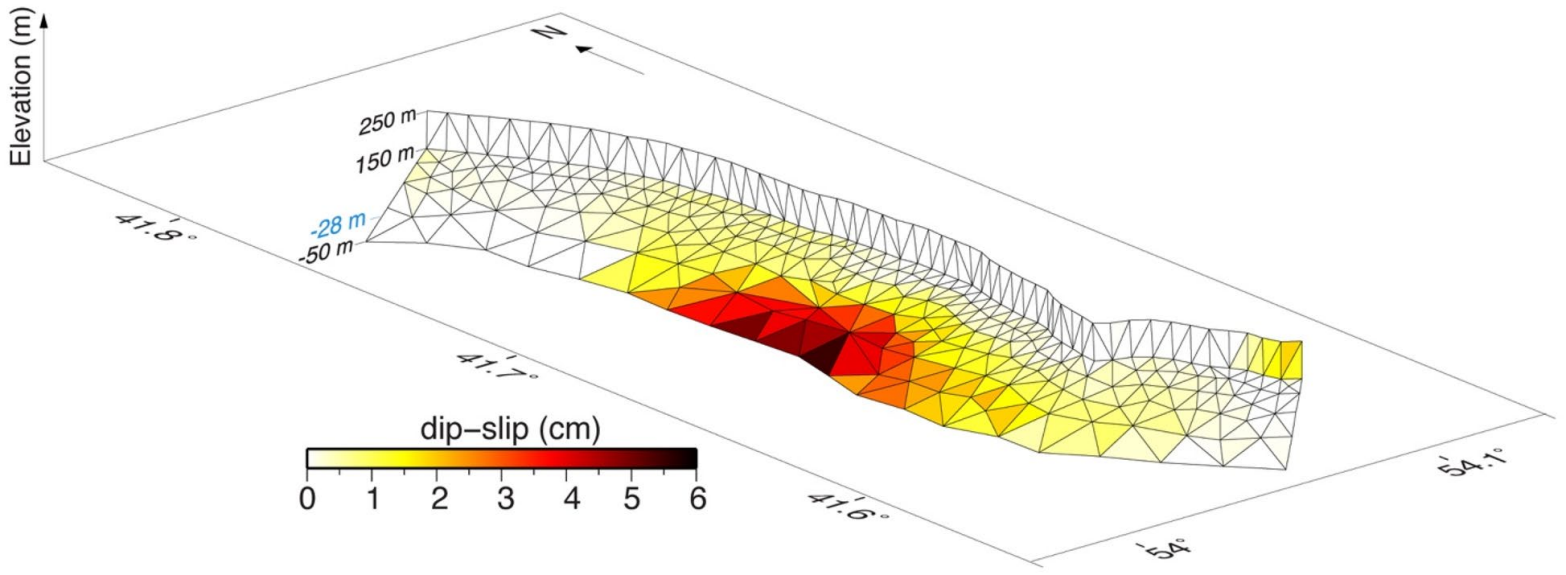
3D view with 1:8 vertical exaggeration of the modelled near flat décollement (~ 2°) connected to the steeper scarp (60°) at the crown of the landslide with the best-guess slip distribution. The − 28 m value shown in blue on the vertical axis indicates the water level. Figure generated using Generic Mapping Tools (GMT v5.3.1; http://gmt.soest.hawaii.edu/).

We performed inversions for the distributed slip on a nearly flat décollement (~ 2°) connected to the steeper scarp (60°) at the crown of the landslide planes to provide a slip surface that effectively approximated the observed InSAR data. As expected, most of the slip was concentrated at the lower part of the slip surface. The maximum slip occurred at a depth of 50 m. Our model, supported by the goodness of fit to the observation (Supplementary Figs. 4–6), suggests that motion on a planar surface at shallow depth can explain the InSAR observations very well (Fig. 5).

**Figure 6:**
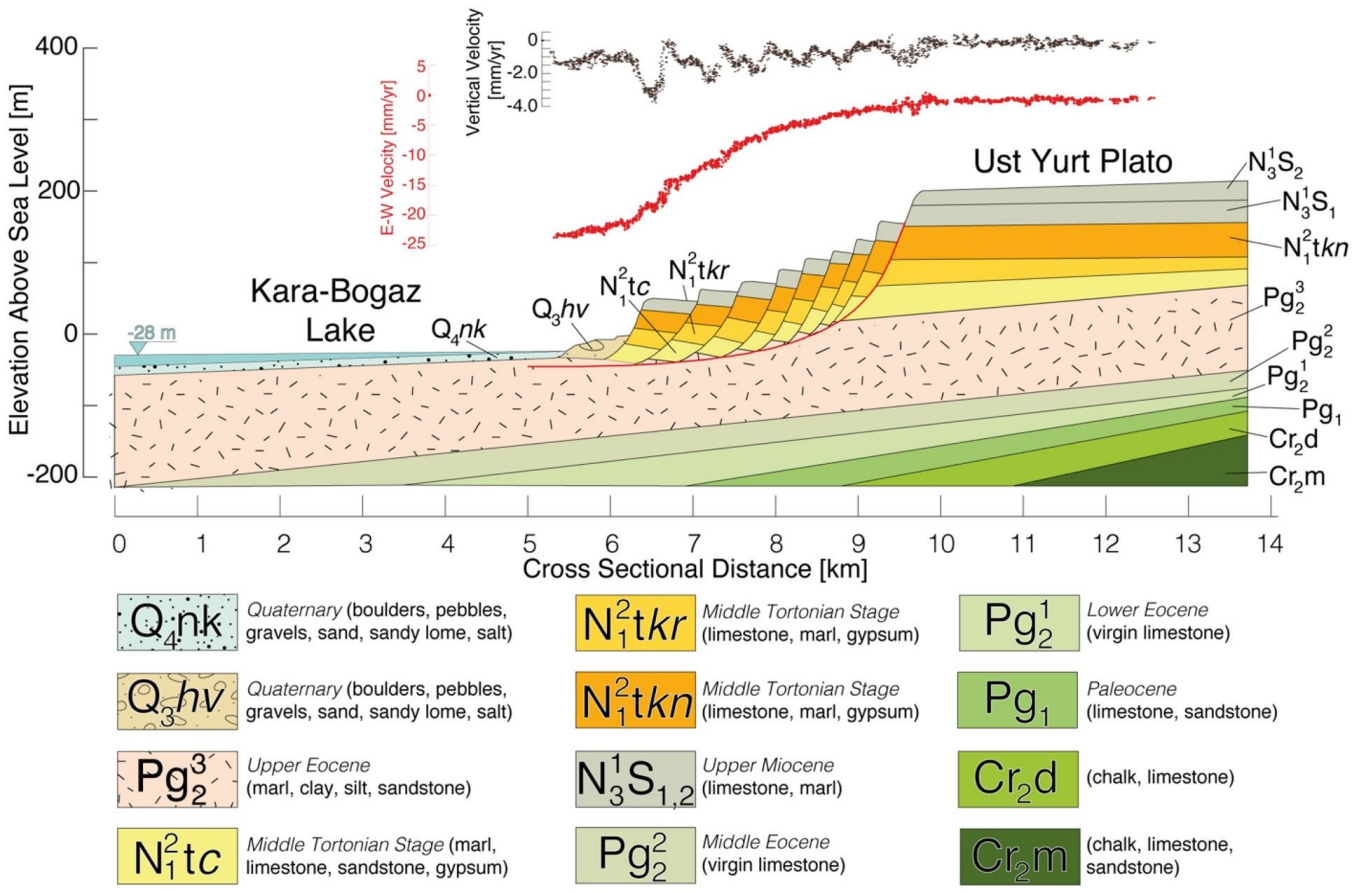
Schematic diagram of the geological cross-section (A–B) of the landslide including horizontal (red) and vertical (black) velocities. The geological map, cross-section, and lithological descriptions were obtained from the OneGeology Platform (http://www.onegeology.org). Line A–B in Fig. 1c shows the profile location. This figure was created with Adobe Illustrator CS6 ver. 16 (http://www.adobe.com/products/illustrator.html).

The geology beneath the landslide (Tuarkyr unit) is composed of limestone, sandstone, marl, and gypsum deposited approximately 7–11 mya over the upper Eocene unit consisting of silt, clay, marl, and sandstone deposited about 35 mya. As shown in Fig. 6, the geological formations associated with the landslide created an area of weakness at the contact between the competent limestone layers and the overlying clay layer. Figure 6 shows the vertical and horizontal velocity profiles along the geological cross-section, revealing competent limestone overlying weak and plastic claystone lithology in contact with the lake water. This suggests that the seasonal increase in water level might contribute to the increase in pore water pressure at the contact, which could explain the seasonal transient horizontal motion along the shear surface.

## Discussion and conclusions

It has been suggested that the timing of the initial catastrophic failure of the KBG megaslide matched that of CS water-level highstands in the late Pleistocene. Moreover, it is widely accepted that climatic factors are some of the most important triggers of mass movement; therefore, they have been used to explain the temporal occurrence of ancient landslides. The present study is the first to present evidence proving that Pleistocene-triggered geomorphic responses to sea-level changes of the CS remain currently active. A section of the eastern bank of the KBG lagoon that is ~ 25 km long by ~ 5 km wide is sliding toward the lake at a rate of 25 mm/year, with a clear dominance of horizontal motion on a shallow, gently dipping (2°), listric detachment plane. Slow vertical motions with up to 4 mm/year subsidence (as shown in the vertical velocity profile in Fig. 6) coincide with the successive and highly regularly-spaced steep scarps that we interpret as potential fault planes where rotational sliding occurs. The absolute altitude of the limestone blocks decreases in the direction from the cliff to the coast of the reservoir in a very regular fashion that resulted in a stair-step topography. The proposed mechanism consists of the break-down of blocks of competent limestone that lose adhesion to the main limestone layer covering the Ustyurt plateau and rotates over the weak clays-rich layers. In this study, we highlighted transient episodes of motion superposed to a constant creep.

Analysis of remotely sensed SAR data acquired from multi-viewing geometries and PSI data allowed us to extract both horizontal and vertical motions of the slowly moving giant landslide. To characterize the kinematics of this mega landslide, we compared the multi-annual time series of InSAR data with the time series of key factors that control the hydrological forces in the study area, such as the water level in the KBG reservoir, soil moisture, temperature, and rainfall. Our results suggest that such an immense mass movement is not currently stable; rather, seasonally significant acceleration occurs annually in accordance with the maximum water level of the KBG reservoir and soil moisture. The strong correlation between the mega landslide movement episodes and the reservoir water level shows its sensitivity to present-day hydrological forcing. This indicates a strong influence of pore water pressure variation on the kinematics of this massive slope failure. The results show how the present-day giant slope instability is affected by changes in climate and the hydrological balance occurring remotely, which originated during successive CS transgressions in the Late Glacial. This giant slope failure occurs presently in a dry, low-gradient region that is otherwise not susceptible to major landslide events. Moreover, given the active seismicity in the region, liquefaction might follow shear compaction, or it can be induced by strong earthquakes. Such movement is another likely trigger of regional slope instability that requires further investigation.

The effects of past climate change on terrestrial landslides in the Quaternary context have been strongly debated. However, the present study presents new information on the potential role of ancient landslides on present-day climate change and sea-level rise.

Future study of the relationships between landslide kinematics and sea-level changes in the CS and the KBG will benefit by combining X-band SAR data with ground-based SAR interferometry to derive spatial and temporal high-resolution deformation fields of this giant mass movement. The results of such research can be used to precisely explore the dimensions and detachment plane geometry and to further detail potential geohazards and environmental interactions.

## Methods

### SAR datasets

The Copernicus Sentinel-1 C-band SAR mission is a joint initiative of the European Commission (EC) and the European Space Agency (ESA), which operates the Sentinel-1A and -1B twin space-borne segments. The Sentinel-1 Terrain Observation with Progressive Scan (TOPS) mode offers important advantages compared with that of other sensors because it provides wide area coverage and a short revisit time of 6 days over Europe and 12 days globally. The temporal resolution of the Sentinel 1-A dataset increased from 12 days until October 2016 to 6 days after the launch and beginning of the operational phase of its twin satellite Sentinel 1-B. Sentinel-1 data are free and openly accessible via various sources; we used the Copernicus Open Access Hub (http://scihub.copernicus.eu) to obtain this information. The wavelength is 5.547 × 10^–2^ m, and the single-look pixel spacing in the azimuth and range directions is 13.9 m and 2.3 m, respectively. In the present study, we processed three independent sets of Sentinel-1A/B imagery acquired along descending and ascending orbits (Track 57 [Asc], Track 35 [Dsc], and Track 137 [Dsc]) to map the surface displacement field of the slow-moving coastal landslide along the 20-km-long strip of the eastern bank of the KBG. The study area is covered entirely by all tracks (Fig. 1a). On the ascending track 57, we computed 118 interferograms; on descending tracks 35 and 137, we formed 130 and 103 pairs, respectively, as detained in Supplementary Table 1 and Supplementary Fig. 5. In addition, we employed individual sets of interferograms for each track with sufficiently high phase coherence patterns over the study area. The region examined here is completely arid with fairly flat topography and no vegetation, which provided favorable image coherency for interferometric SAR processing. The average PS density calculated from the three different datasets over the entire area of interest was 400 points/km^2^.

### PSI processing methodology

All interferograms were generated using the Generic Mapping Tools Synthetic Aperture Radar (GMTSAR) open source InSAR processing system^40^. To correct the topographic contribution to the radar phase, we used the Shuttle Radar Topography Mission (SRTM) 3-arcsecond digital elevation model^41^. All interferograms were computed based on a single master network for PSI analysis. The choice of the master images minimized the spatial and temporal baselines. The single master stacks of interferograms were processed using the STAMPS software package^18,19^, which enabled identification of the PS points using both amplitude and phase information. In the first step, the initial selection of the PS points was performed on the basis of their noise characteristics using amplitude analysis. The amplitude dispersion criterion is defined by

1 $$D_{amp} = \left( {\sigma_{Amp} /m_{Amp} } \right),$$

where $\sigma_{Amp}$ and $m_{Amp}$ are the standard deviation and mean of the amplitude in time, respectively^42^. We selected a threshold value of 0.27, which minimized the random amplitude variability and eliminated the highly decorrelated pixels in some areas, such as those covered by vegetation, agricultural fields, or snow. Once the stable PS targets were selected based on amplitude analysis, the PS probability was refined by phase analysis in a series of iterations. This process enabled detection of stable pixels even with low amplitude. Once the final PS selection was conducted, the residual topographic component was removed. Then, phase unwrapping was performed both spatially and temporally. This analysis enabled retrieval of the average LOS surface deformation rate maps. To remove the atmospheric effects from the interferograms, we used the freely available Toolbox for Reducing Atmospheric InSAR Noise (TRAIN)^43^. This toolbox uses ERA-Interim (ERA-I, European Center for Medium-Range Weather Forecast) numerical weather model datasets^44^.

### Deformation decomposition into 2D displacement rates

The InSAR technique measures the projections of ground displacement along only the LOS look angle. To retrieve the components of actual deformation, the InSAR LOS displacement fields observed under different viewing directions (i.e., different satellite tracks) over the same ground location must be combined. We decomposed the mean PSI LOS velocity fields into E–W and vertical components by neglecting the motion along the N–S direction, which is a reasonable assumption for motion on a west-facing slope. We used only the LOS mean velocity fields calculated from the Sentinel 1 A/B images, which cover the same time interval. Initially, we resampled the mean LOS velocities for the ascending and descending tracks onto a similar grid with 100‐m pixel spacing using a nearest neighbor procedure to resample the persistent scatterer pixels within 200 m of the center of each grid nodal point. In the second step, we selected the pixels present in both velocity maps of the ascending and descending tracks. Before decomposition, we transformed the InSAR mean velocity fields of both tracks into the same reference frame using a reference area considered to be a stable area, as shown by the black point in Fig. 2. Finally, the LOS velocity fields were decomposed into two components. As a result, the horizontal component along the E–W direction ($d_{hor}$) and the vertical component ($d_{ver}$) were computed considering the local incidence angle of the satellite view by solving the following equation^45^:

2 $$\left( {\begin{array}{*{20}l} {d_{asc} } \\ {d_{dsc} } \\ \end{array} } \right) = \left( {\begin{array}{*{20}c} {cos\theta_{asc} } & \quad { - cos\alpha_{asc} sin\theta_{asc} } \\ {cos\theta_{dsc} } & \quad { - cos\alpha_{dsc} sin\theta_{dsc} } \\ \end{array} } \right)\left( {\begin{array}{*{20}c} {d_{ver} } \\ {d_{hor} } \\ \end{array} } \right),$$

where $\theta_{asc}$ and $\theta_{dsc}$ represent the local incidence angles and $\alpha_{asc}$ and $\alpha_{dsc}$ are the satellite heading angles in the ascending and descending modes, respectively.

### AIC

Several methods are used to test the model fit for a given set of data. The AIC^38^ is a fined technique that estimates the goodness-of-fit of each model relative to that of the other models. When used alone, however, the AIC score of a particular model for a given dataset is not useful unless it is compared with the AIC score of the competing model. Therefore, it is highly useful to compare the absolute likelihood of the model’s fit to the data. The following equation is used to estimate the AIC of a model:

3 $$AIC = - 2 \times {\text{ln}}\left( L \right) + 2 \times k,$$

where $L$ is the maximum value of the likelihood function for the model, and $k$ is the number of estimated parameters in the model. The model that minimizes the $AIC$ score among all other models for a given set of data is the preferred model. $AIC$ scores are often shown as $\Delta AIC$ scores, such as that used in our study, which signifies the difference between the best model with the smallest $AIC$ score and that of a competing model^46^.

In our case, the $AIC$ score was first independently computed for each time series for a model of linear velocity (model 1) and a transient model (model 2). The $AIC$ difference between the two models ($\Delta AIC$) reflects the most robust model (Fig. 4b), with a positive difference indicated when the transient model is preferred. We then summed the $\Delta AIC$ scores of the three independent time series (Fig. 4c).

### Modeling InSAR data

To investigate the manner in which a model of sliding plane geometry can explain the InSAR measurements, we used a 3D slip-inversion method based on the analytical dislocation for an angular dislocation in a linear elastic, homogeneous, isotropic half-space with damped least-squares minimization. We acknowledge that this gigantic landslide deformation is nonrecoverable, but we justify the use of the linear elastic dislocation model to infer landslide subsurface slip geometry for the following reasons. First, the surface displacements and the strains are relatively small in our case. In such cases, a linear elastic model assumption can be a fair approximation when the first-order linear term in the strain tensor is much larger than the higher-order nonlinear terms. Second, in the interest of exploring the transient motion characteristic of the observed landslide modulated by hydrological forces, rather than delivering the best characterization of the siding surface, we are willing to trade the imperfect linear elastic dislocation mechanical assumptions for its computational efficiency and ease of implementation in an inverse approach. Considering the lack of sufficiently detailed rheological information across the Ustyurt Plateau, another significant advantage of the present method lies in the fact that few constitutive parameters are required as input data and that most of these parameters can be readily obtained by conventional geotechnical tests. Our inverse modelling is based on the boundary element method^47^ that employs a set of planar triangular elements of constant displacement discontinuity to model the dislocation plane resembling the landslide basal décollement and the first slip plane that exposes at the crown area. When the décollement dipped eastward at an angle of ~ 2° from depths of 150 to 350 m, the main scrap at the crown dipped between 50° and 60° and joined the décollement at depth. To avoid unphysical oscillatory slip, the scale-dependent umbrella smoothing operator of the Poly3Dinv code was applied to the inverted slip distribution with a factor of 0.25^48^. In the inversion, dip slip was inverted only when no strike-slip component was present. Modelling explained the LOS velocity fields with an RMS misfit of about 1 mm. The results shown in Supplementary Figs. 4, 5 and 6 confirm the observations of many secondary slip planes that also displaced the landmass above the décollement; however, they were negligible.

## Supplementary Information

Supplementary Information.
