# Supplementary material for: Transient motion of the largest landslide on earth, modulated by hydrological forces
Source: Sci Rep. 2021 May 17;11:10407. doi: 10.1038/s41598-021-89899-6 (PMC8129149; doi:10.1038/s41598-021-89899-6)
Supplement: Supplementary file 1 — Supplementary Information. [file 41598_2021_89899_MOESM1_ESM.docx]

**Supplementary Information for**

Transient Motion of the Largest Landslide on Earth, Modulated by Hydrological Forces

Gökhan Aslan^1, *^

Marcello De Michele^1^

Daniel Raucoules^1^

Severine Bernardie^1^

Ziyadin Cakir^2^

^1^Natural Risk Department, BRGM—French Geological Survey, 3 Claude-Guillemin 45060 Orléans, France

^2^Department of Geological Engineering, ITU, Istanbul 34467, Turkey

^*^Correspondence to Gökhan Aslan, gokhanaslan3@gmail.com

**This file includes:**

Supplementary Figures 1-6

Supplementary Tables


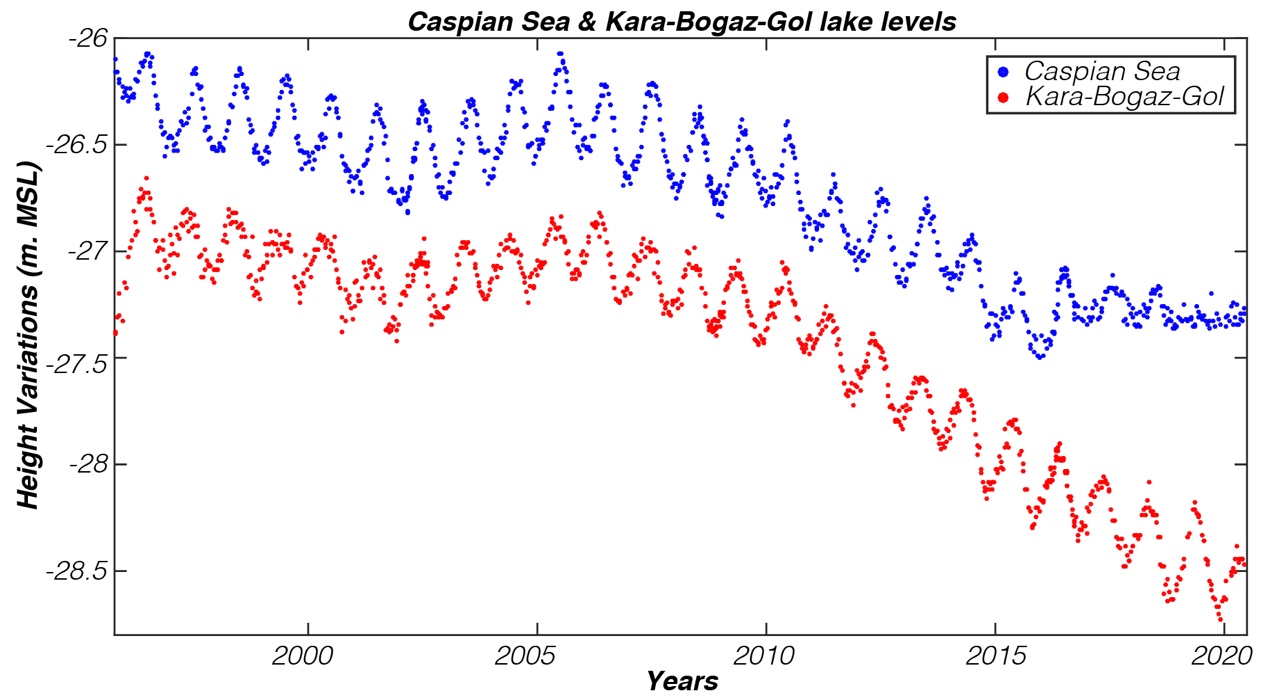
**Supplementary Figure 1 |** Interannual and seasonal variability of Caspian Sea (blue) and Kara-Bogaz-Gol Bay (red) absolute level in meter above mean sea level (MSL) obtained from TOPEX/POSEIDON and Jason series Altimetry provided by the USDA Global Reservoir and Lake Elevation Database, (<https://ipad.fas.usda.gov/cropexplorer/global_reservoir>). This figure was created with MATLAB (R2011a). (<https://www.mathworks.com/products/matlab.html>).


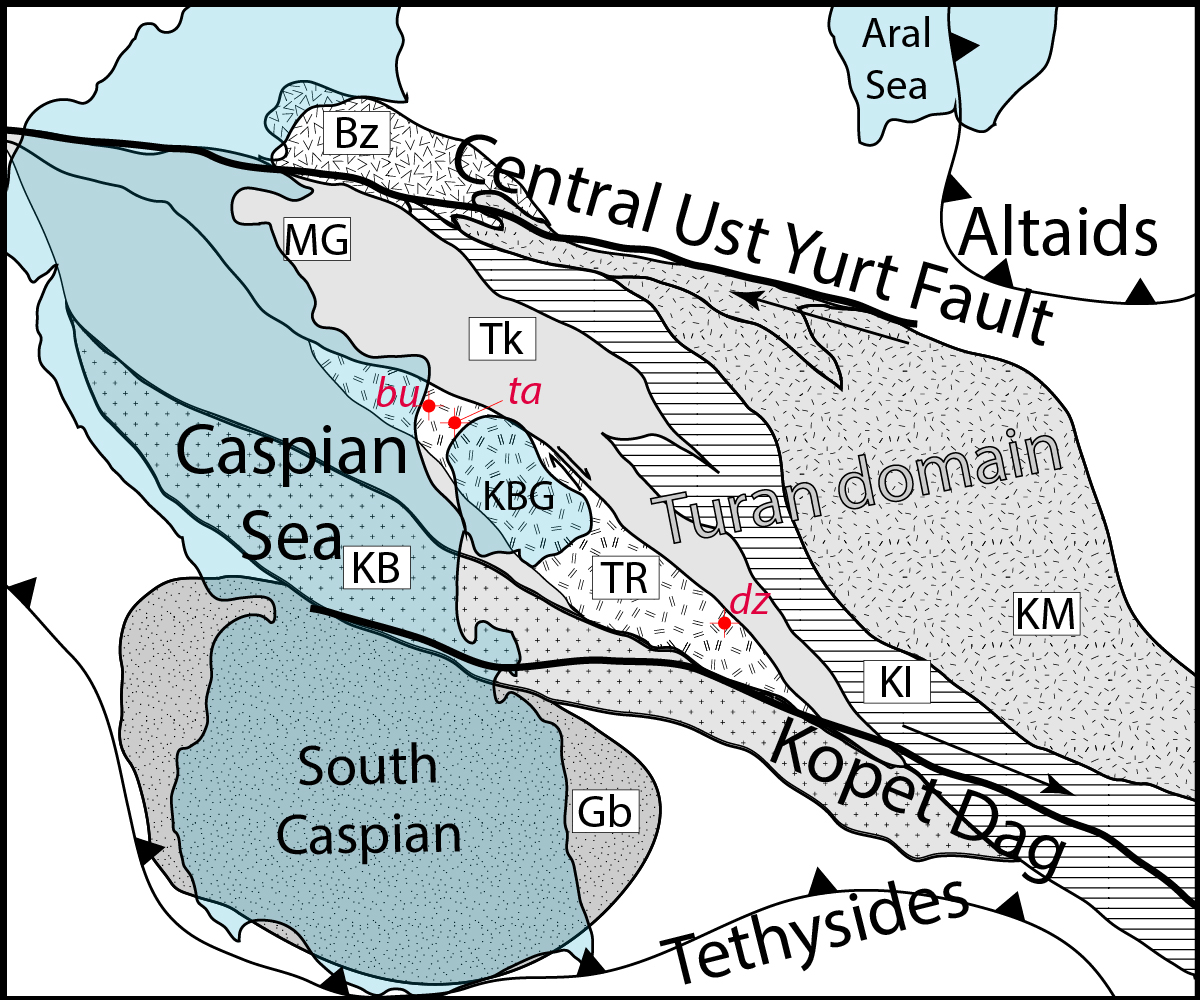


**Supplementary Figure 2** | Tectonic units of the Turan domains bounded by Central Ust Yurt Fault in the north and Kopet dag Fault in the south : KB - Karabogaz Unit; MG - Mangyshlak subunit; TR - Tuarkry unit; KM - Karakum Unit; Tk - Tuarkry - Karaudan Fault; Gb - Great Balkhan Range. Red circles represents the locations of boreholes that penetrate the basement (Mainly from [Bakirov (1970)](#aa1970) ): Jamal well (dz); Bukbash well (bu) and Tamdy well (ta)

This figure was created with Adobe Illustrator CS6 ver. 16 ([http://www.adobe.com/products/illustrator.html](This%20figure%20was%20created%20with%20Adobe®%20Illustrator%20CS6%20ver.%2016%20(http:/www.adobe.com/products/illustrator.html).)).

**Supplementary Table 1. Data coverage from each track used in this study**

| Track | Sensor | \| Vieving \| \| --- \| \| Geometry \| | \| Time \| \| --- \| \| Interval \| | \| Sub-Swath \| \| --- \| \| Number \| | \| Incidence \| \| --- \| \| Angle (°) \| | \| # of Interferograms \| \| --- \| \| Used \| | \| Density \| \| --- \| \| (PS/km^2^) \| |
| --- | --- | --- | --- | --- | --- | --- | --- | --- | --- | --- | --- | --- | --- | --- | --- | --- | --- | --- | --- |
| T57 | SENTINEL | Ascending | 2014-2020 | IW3 | 39.9° | 118 | ~ 410 |
| T35 | SENTINEL | Descending | 2014-2020 | IW1 | 31.6° | 130 | ~ 330 |
| T137 | SENTINEL | Descending | 2014-2020 | IW3 | 42.1° | 103 | ~ 400 |


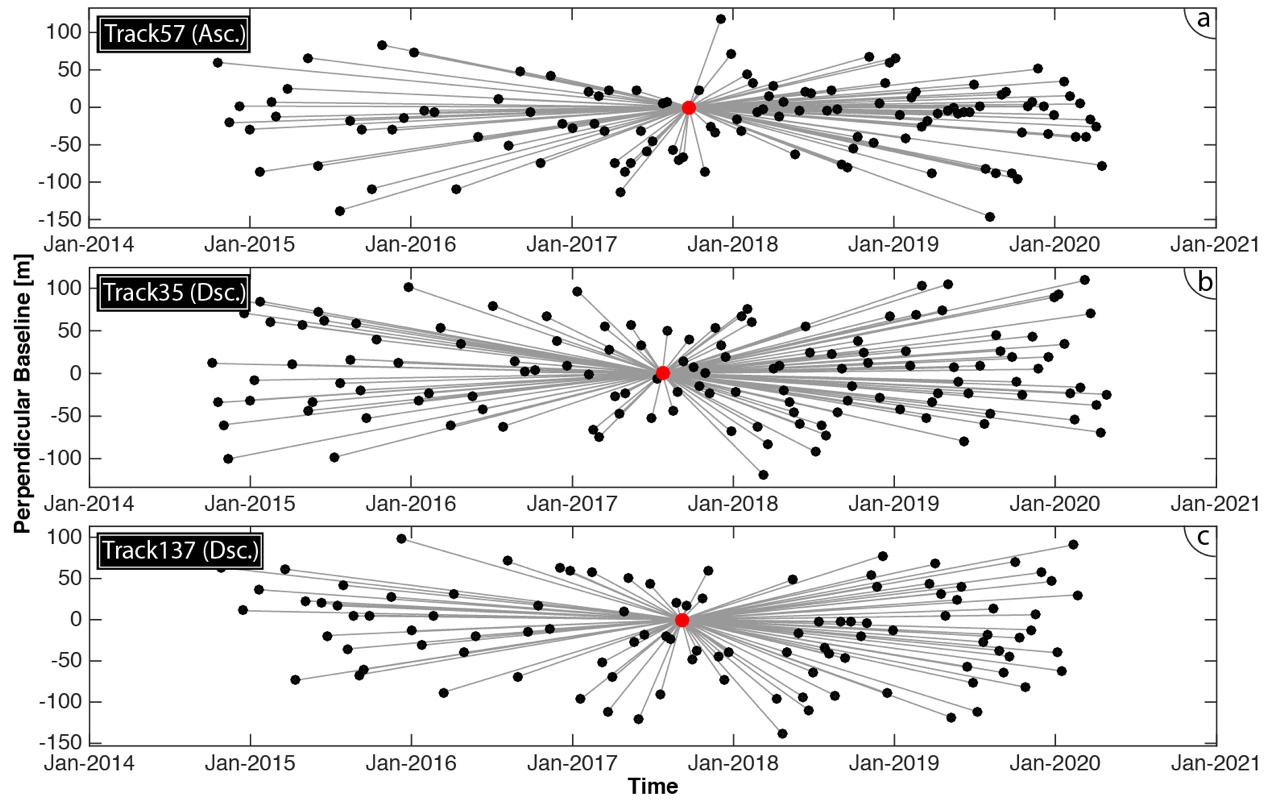
**Supplementary Figure 3 |** Baseline configurations of single-master Interferograms. Baseline versus time plots for Sentinel ascending track 57 and for the descending tracks 35 and 137 used in this study with black dots donating the time of image acquisitions. The red dots indicate the master image used as a reference for each track. Gray lines connect pairs (Interferograms). This figure was created with MATLAB (R2011a). (<https://www.mathworks.com/products/matlab.html>).


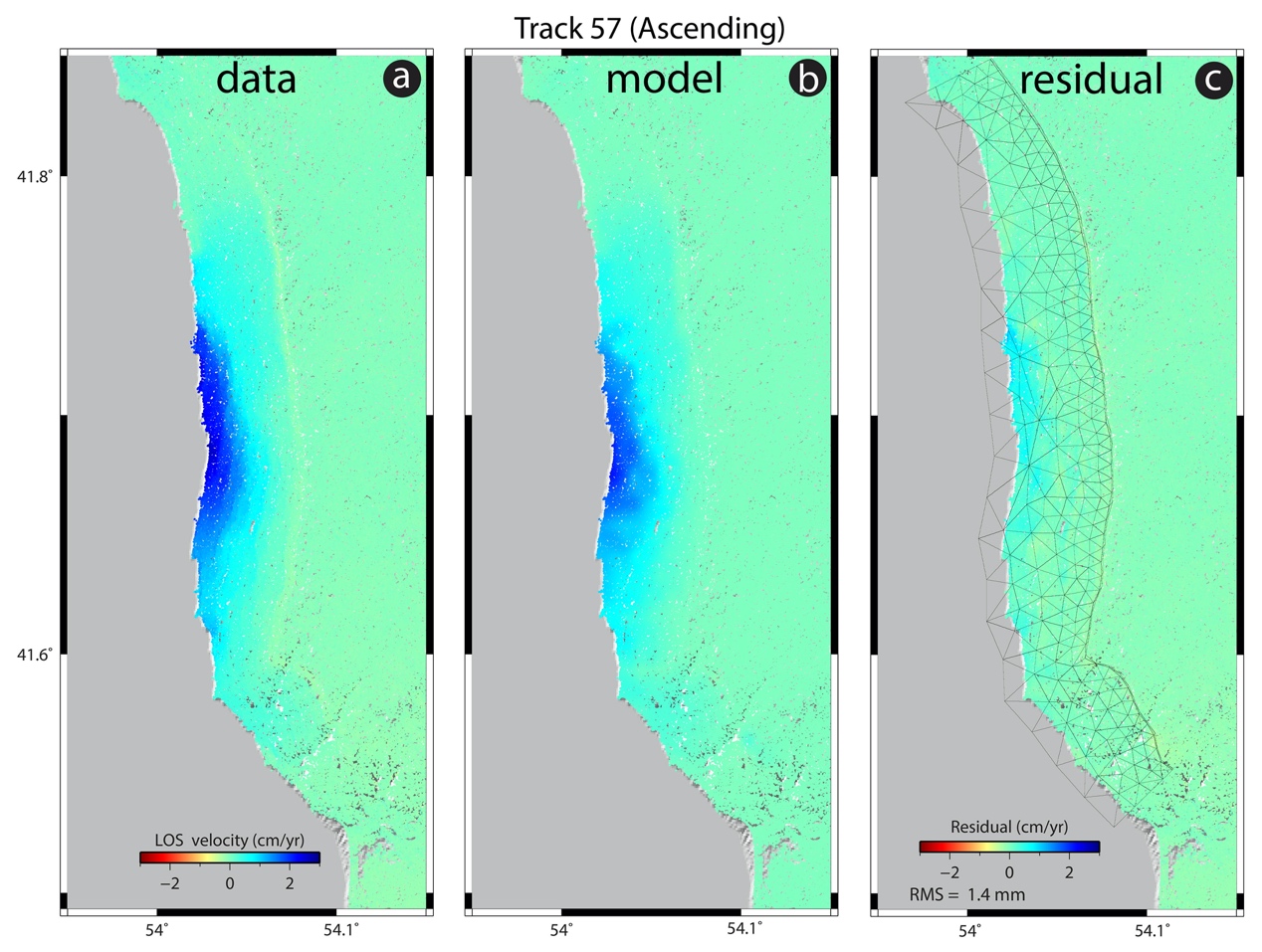


**Supplementary Figure 4 |** Processed InSAR data from ascending track 57 (a), our best fitting model prediction (b) and the model residual after moving model prediction from data (c). Figure generated using Generic Mapping Tools (GMT v5.3.1; <http://gmt.soest.hawaii.edu/>).


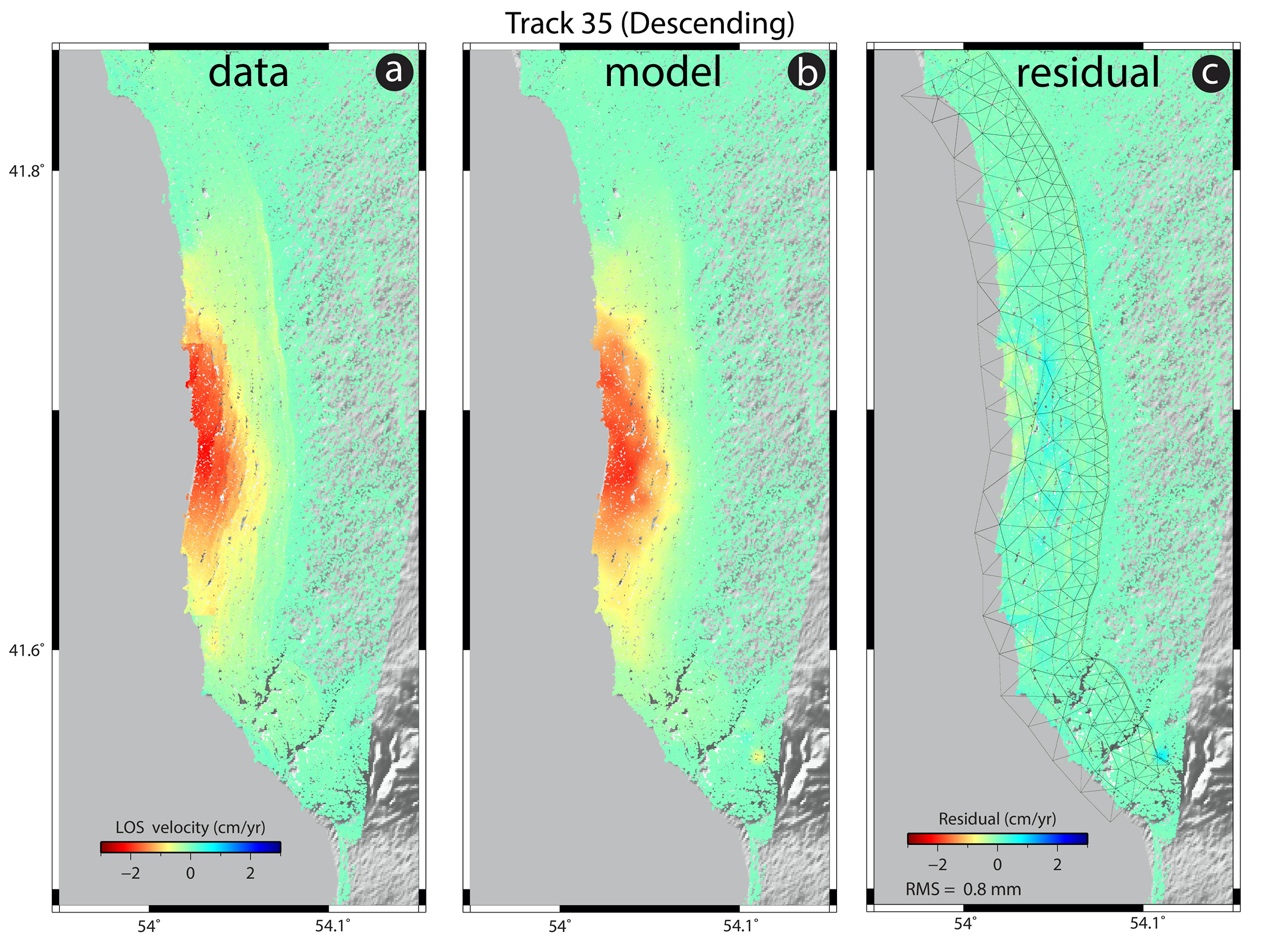


**Supplementary Figure 5 |** Processed InSAR data from descending track 35 (a), our best fitting model prediction (b) and the model residual after moving model prediction from data (c). Figure generated using Generic Mapping Tools (GMT v5.3.1; <http://gmt.soest.hawaii.edu/>).


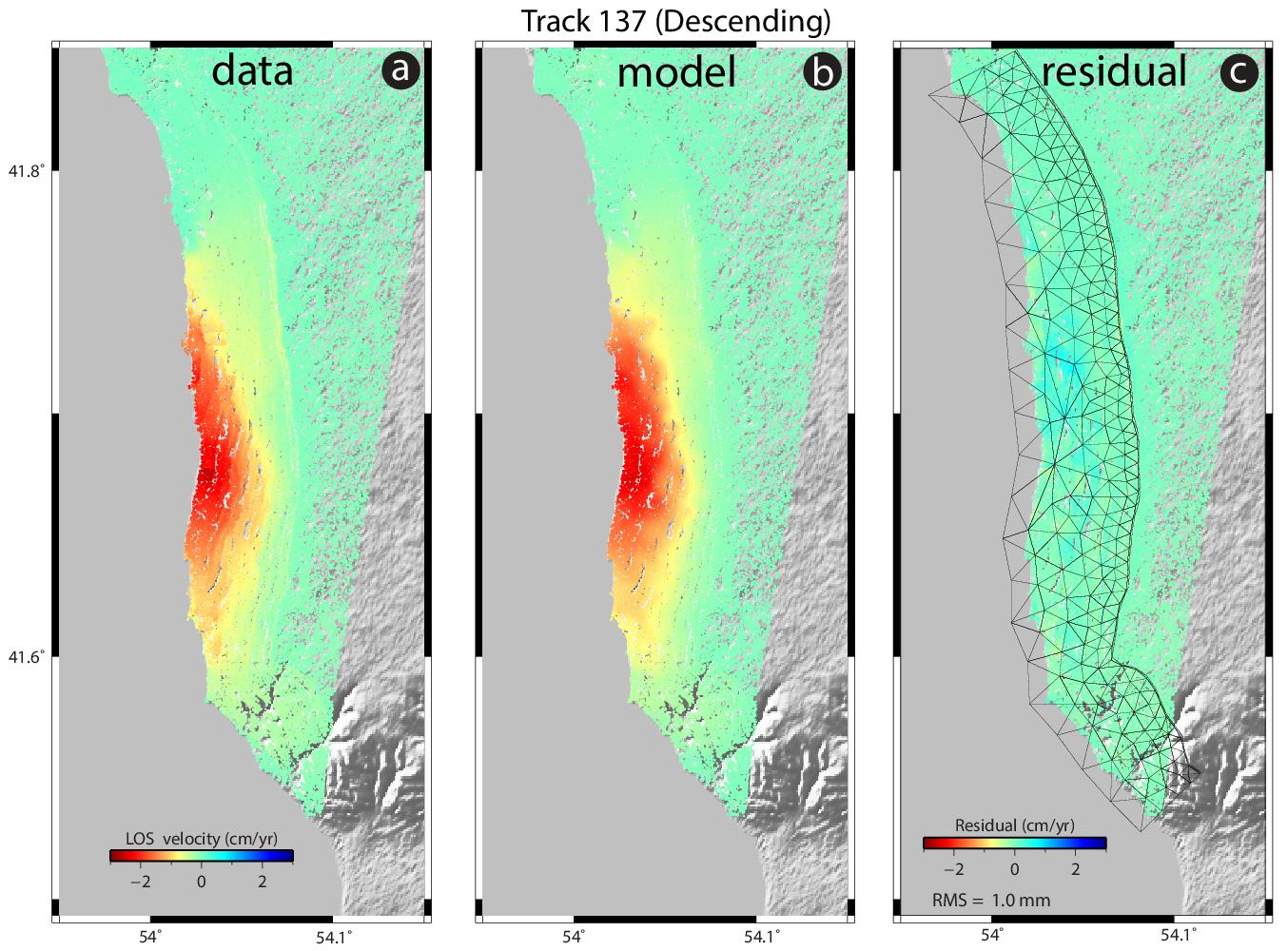


**Supplementary Figure 6 |** Processed InSAR data from ascending track 137 (a), our best fitting model prediction (b) and the model residual after moving model prediction from data (c). Figure generated using Generic Mapping Tools (GMT v5.3.1; <http://gmt.soest.hawaii.edu/>).
